# Bioactive and Molecular Characterization of Mealworm (*Tenebrio molitor*) Powder: Nutritional and Functional Implications for Crackers

**DOI:** 10.3390/ijms27209378

**Published:** 2026-10-21

**Authors:** Ewelina Zielińska, Anna Jakubczyk, Kamila Jaglińska, Beata Polak

**Affiliations:** 1 Department of Analysis and Evaluation of Food Quality, University of Life Sciences in Lublin, Skromna Str. 8, 20-704 Lublin, Poland; 2 Department of Biochemistry and Food Chemistry, University of Life Sciences in Lublin, Skromna Str. 8, 20-704 Lublin, Poland; anna.jakubczyk@up.edu.pl; 3 Department of Physical Chemistry, Medical University of Lublin, Chodźki 4a, 20-093 Lublin, Poland; kamila.jaglinska@umlub.edu.pl (K.J.); beata.polak@umlub.edu.pl (B.P.)

**Keywords:** edible insects, entomophagy, predicted glycemic index, starch digestibility, FTIR analysis, phenolic compounds, texture, consumer acceptance

## Abstract

Edible insects are increasingly recognized as a viable alternative food source, highlighting the urgent need for systematic research into their integration into conventional food products. This study examined the effect of adding mealworm powder (*Tenebrio molitor*) to crackers, focusing on factors such as color, texture, nutritional value, antioxidant properties, starch digestibility, and molecular structure. As the level of mealworm powder increased (from 2% to 10%), the crackers became darker, while instrumental hardness and fracture time decreased, indicating that a more brittle structure formed. Nutritionally, the enriched crackers contained higher protein, fat, and mineral content, while their carbohydrate content decreased; moreover, their protein profile was modified. Antioxidant activity and total phenolic content also rose in proportion to the level of mealworm enrichment. FTIR analysis revealed differences in spectral regions associated with proteins, lipids, and carbohydrates, indicating compositional and local molecular-environment changes following mealworm powder incorporation. Mealworm powder incorporation markedly decreased rapidly digestible starch and increased slowly digestible starch, whereas the predicted glycemic index showed only a modest but statistically significant reduction at higher supplementation levels. Furthermore, sensory evaluation indicated that consumer acceptance was optimal at a low inclusion level of 2%. Overall, mealworm powder represents a promising ingredient for improving selected nutritional and functional characteristics of crackers; however, its inclusion level should be optimized to maintain sensory quality.

## 1. Introduction

With rapid global population growth, increasing pressure on natural resources, and worsening food security, finding alternative, sustainable food sources is becoming a key challenge in modern science. Traditional meat production systems play a vital role in meeting human nutritional needs; however, they require large amounts of water, emit greenhouse gases, and have significant environmental impacts. As a result, there is growing interest in innovative solutions that could provide a more efficient and environmentally sustainable alternative [1].

One of the most promising areas of research is edible insects, which are increasingly being recognized as a valuable source of nutrients. Producing insects for food has a much lower environmental impact than traditional livestock farming. Insects require less feed, water, and space, and their rearing produces significantly lower greenhouse gas emissions. For these reasons, insects are viewed as a promising component of future food systems that support sustainable development [1].

Insects are rich in high-quality protein, essential amino acids, unsaturated fatty acids, vitamins (especially B vitamins), and micronutrients such as iron, zinc, and calcium [2]. Consequently, they could play a significant role in improving dietary quality, particularly in regions affected by malnutrition or limited access to traditional protein sources. In addition to their high nutritional value, edible insects are increasingly recognized as a source of biologically active compounds that offer potential health benefits. Research has identified various such molecules, including phytosterols, polyphenols, antimicrobial peptides, chitin and its derivatives, and bioactive lipids [3]. These compounds may contribute to disease prevention and overall health improvement. Polyphenols exhibit strong antioxidant properties, helping to neutralize free radicals and reduce oxidative stress linked to chronic diseases such as cancer and diabetes. Their effectiveness may be enhanced through interactions with other insect-derived compounds [4]. It is important to note that the biological activity of insect-derived compounds can be influenced by processing methods and food formulation [3]. For example, incorporating insect flours into food products can lead to interactions with other ingredients, enhancing antioxidant capacity and affecting starch digestibility and glycemic response [5]. Overall, these bioactive compounds significantly expand the role of edible insects beyond basic nutrition, positioning them as functional food ingredients that can support health and prevent disease.

In recent years, interest in using insects as ingredients in various food products has grown. Scientific research has focused on incorporating insect powders, such as mealworms (*Tenebrio molitor*) and house crickets (*Acheta domesticus*), into foods like baked goods [6], pasta [5,7], extruded snacks [8], protein bars [9], cookies [10], and burgers [11]. Studies have shown that adding these insect powders significantly increases protein content, improves the amino acid profile, and enhances mineral content in these products. Additionally, the use of insect ingredients can influence the food’s technological and sensory properties [4]. However, it is important to optimize recipe compositions to ensure that the final products maintain acceptable organoleptic qualities. Although edible insects offer many benefits, their acceptance in the daily diet—especially in European countries—faces cultural and psychological barriers. As a result, an important area of research is focused on developing products that incorporate insects in a way that is not directly visible, while still serving as functional ingredients that enhance nutritional value [12].

This paper examines mealworm-powder-enriched crackers, showcasing an innovative product with potential functional benefits. Unlike previous research, this study is among the first to systematically evaluate the multidimensional effects of adding insect powder across various aspects, including the product’s nutritional value, starch digestibility, predicted glycemic index, and antioxidant activity. This comprehensive approach enables a more thorough assessment of how this ingredient affects food quality. Additionally, the study includes an analysis of color, texture, and sensory characteristics, all of which are crucial for consumer acceptance and serve as the foundation for developing an optimal formulation for the new product. Another significant innovation is identifying bioactive compounds in insect powder, which may enhance the crackers’ potential functional properties.

The proposed approach aligns with current research trends in functional foods and alternative protein sources, while also providing new data on the potential of using insects to produce high-value, nutrient-rich products with beneficial physiological properties.

Although several previous studies have investigated insect-enriched bakery products, including crackers and other baked goods, most have focused primarily on selected quality parameters, such as nutritional composition, sensory acceptance, or technological properties. In contrast, our study provides a comprehensive, systematic evaluation of mealworm powder incorporation by simultaneously assessing its effects on nutritional value, starch digestibility, predicted glycemic index, antioxidant activity, color, texture, and sensory characteristics. This multidimensional approach provides a more holistic understanding of mealworm powder’s impact on both technological and health-related aspects of product quality.

Furthermore, this work identifies and characterizes bioactive compounds present in mealworm powder and discusses their potential contribution to the functional properties of the enriched crackers. To the best of our knowledge, studies that integrate the assessment of bioactive compounds, antioxidant potential, starch digestibility, and consumer-relevant quality attributes within a single cracker formulation remain limited. Therefore, our research extends current knowledge on insect-enriched bakery products and provides new insights into developing nutritionally enhanced, functionally valuable, and consumer-acceptable foods based on alternative protein sources.

## 2. Results and Discussion

### 2.1. Color Measurements

**Figure 1:**
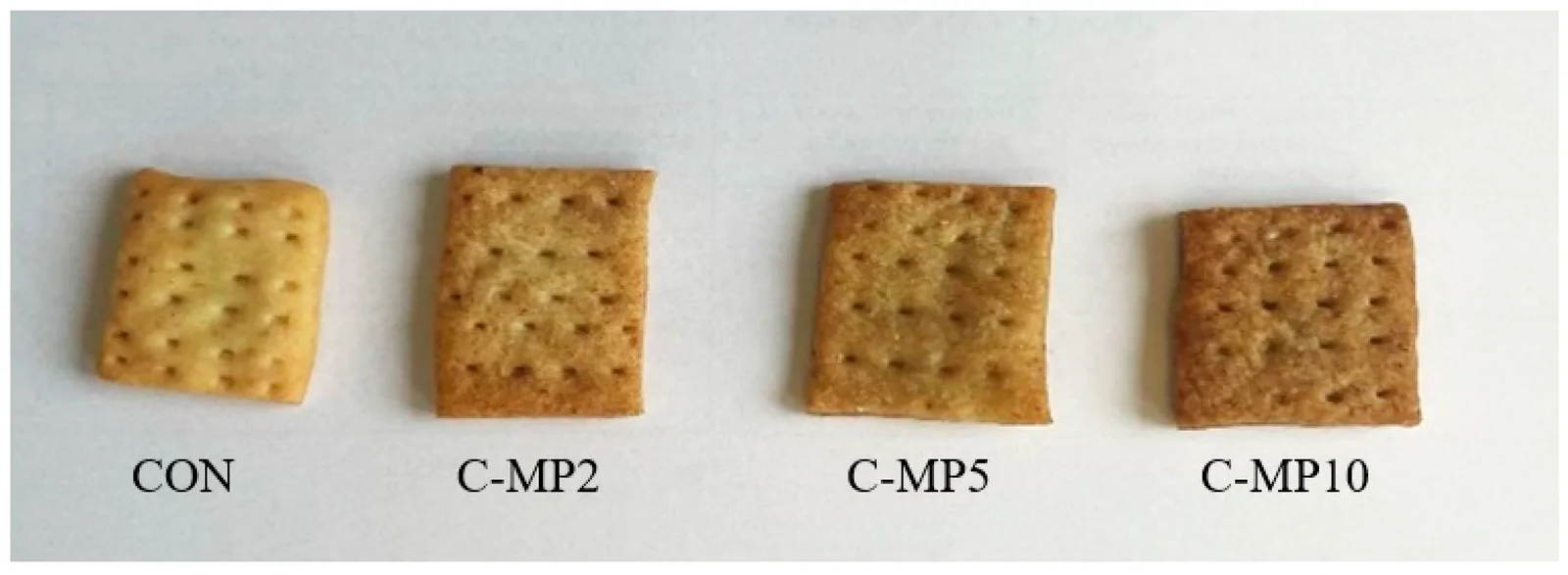
Appearance of crackers. CON—control sample, C-MP2—sample with 2% mealworm powder addition, C-MP5—sample with 5% mealworm powder addition, C-MP10—sample with 10% mealworm powder addition.

The color characteristics of the crackers were assessed using the CIELab color system (L*, a*, b*) (Table 1). This system provides insights into lightness and the red and yellow color components, as well as the total color difference (ΔE) and the browning index (BI). The data clearly indicate that the mealworm powder (MP) additive significantly affects the product’s visual properties (Figure 1).

As MP content increased, the L* value decreased systematically and significantly (*p* < 0.05), indicating that the product gradually darkened—from 78.01 in the control sample to 55.60 in the C-MP10 sample. Concurrently, the a* value increased, indicating a more intense red hue, especially in samples with higher MP content (C-MP5 and C-MP10). In contrast, the b* parameter showed no statistically significant differences (*p* ≥ 0.05), suggesting the yellow component remained relatively stable regardless of the added MP level.

The total color difference (ΔE) increased with higher MP content, ranging from 5.60 for the C-MP2 sample to 21.02 for the C-MP10 sample. Previous studies have indicated that consumers can visually notice color differences when ∆E is greater than 3.0 [13]. This indicates that the color changes were clearly visible compared to the control sample. Additionally, the browning index (BI) rose with the amount of MP added, increasing from 50.91 to 89.01. This suggests an intensified browning processes, such as the Maillard reaction or caramelization.

The color change in baked goods, such as bread, muffins, and crackers, is influenced by several factors. Key among these are baking conditions—specifically, baking temperature and time—as well as the chemical composition and natural colors of the ingredients used. The surface color of crackers is primarily determined by the Maillard reaction, which occurs during baking when proteins react with reducing sugars. When wheat flour is partially replaced with insect flour, the resulting product tends to be darker for two main reasons. Firstly, insect flours are naturally darker in color compared to wheat flour. The brown colour of the mealworm powder is attributed to melanin, the primary pigment [14]. Secondly, they contain a significantly higher protein content. Therefore, it can be concluded that a higher proportion of protein-rich ingredients enhances the intensity of the Maillard reaction.

These changes are crucial to consider, as they affect consumers’ perceptions of product quality. Consumers tend to associate darker-colored products with whole-grain options, which are regarded as healthier choices by health-conscious individuals [15].

The present results are similar to those obtained by Djouadi et al. [16] for crackers with mealworm powder, as well as other authors for different products, e.g., muffins [17], shortcake biscuits [10], bread [18], pasta [5].

### 2.2. Texture

Texture characteristics in crackers are crucial quality attributes because they influence consumer perceptions of crispness and hardness. Hardness refers to the force required to break a cracker. Crackers that are too hard are difficult to bite into, while those that are too soft feel stale. The optimal hardness gives the consumer a satisfying “crunch”. Crispness is the sensation experienced when biting into food. Consumers typically associate it with product freshness, as a lack of crispness often indicates the food has become soggy. Crispness also depends on the ingredients used in the recipe and the preparation method. Crispness is related to the time required to break a cracker.

**Figure 2:**
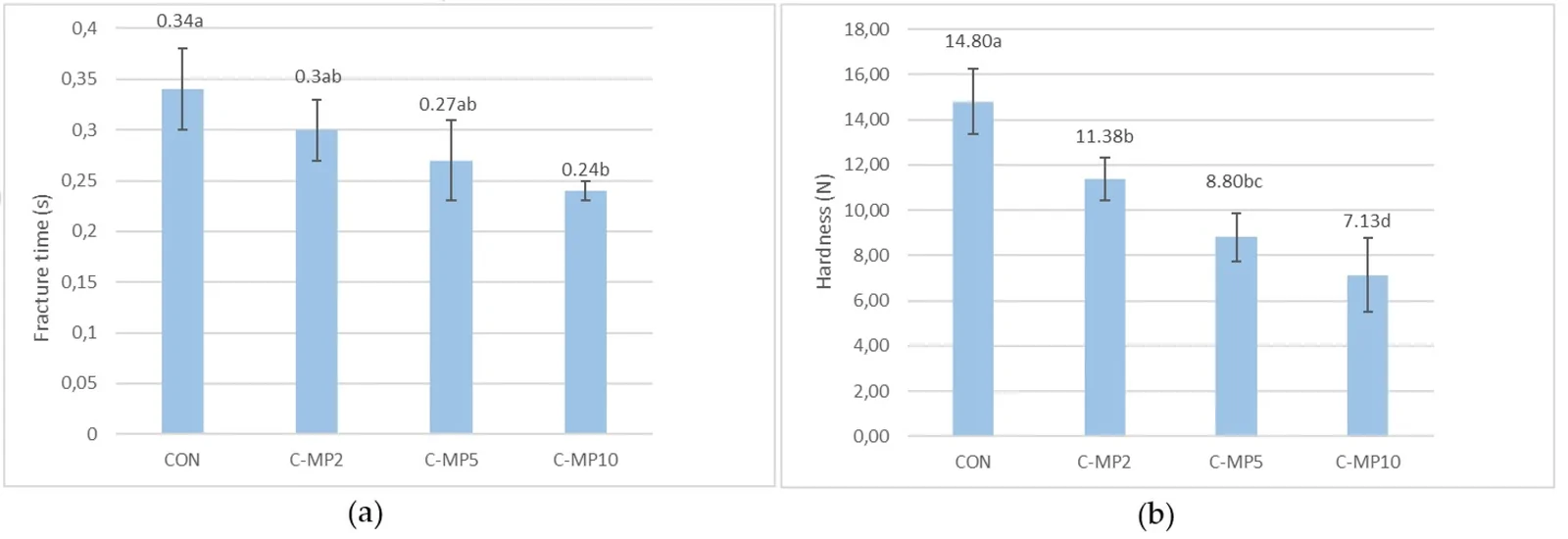
Instrumental texture parameters of crackers: (**a**) fracture time (s); (**b**) hardness (N). CON—control sample, C-MP2—sample with 2% mealworm powder addition, C-MP5—sample with 5% mealworm powder addition, C-MP10—sample with 10% mealworm powder addition. Values with different letters are significantly different (*p* ≤ 0.05)

The texture parameters of the analyzed crackers demonstrated a clear dependence on the level of mealworm powder addition, which affected both the fracture time and hardness of the product (Figure 2). Instrumental fracture time decreased gradually as MP content increased. The control sample exhibited a fracture time of 0.37 seconds, while the C-MP10 variant with a 10% MP addition recorded only 0.24 seconds. This indicates that the product became more susceptible to breaking as MP content increased. Additionally, the sample with a 5% MP addition differed significantly from the control, achieving a break time of 0.26 seconds. The reduction in fracture time indicates that crackers become more susceptible to fracture as MP incorporation increases. This may be associated with changes in matrix continuity caused by partially replacing wheat flour with mealworm powder; however, microstructural analysis would be required to determine whether changes in porosity contributed to this effect.

A similar trend was observed in hardness, defined as the maximum force required to deform a specimen. As the MP content increased, this parameter decreased significantly, dropping from 17.55 N (CON) to 6.39 N (C-MP10). The differences among all analyzed variants were statistically significant (a–d), confirming the substantial influence of the MP additive on the product’s mechanical properties. A reduction in hardness of over 60% indicates a significant weakening of the cracker structure, which may result from decreased interactions between the structure-forming components and diminished cohesion within the matrix.

A combined analysis of the two parameters reveals a consistent mechanism driving structural changes. Increasing MP incorporation significantly reduced both hardness and fracture time, indicating lower resistance to mechanical deformation and more rapid fracture of the crackers. These instrumental changes demonstrate altered mechanical properties of the product but should not be directly equated with sensory crispness. These changes may be linked to loosening of the structural network and disruption of solid-phase continuity, thereby facilitating easier crack initiation and propagation in the material. Similar results were observed in the study by Djouadi et al. [16] on the enrichment of crackers with *T. molitor* powder. They observed that decreased cracker hardness was accompanied by a shorter time to fracture. The presence of mealworm powder also affects the texture of other baked goods; for example, it reduces the hardness of bread [19,20], sponge cake [21] or muffins [17].

From a technological perspective, the results are crucial to the product’s sensory quality. Increased crispness can be desirable, as it enhances the “crunchiness” consumers often appreciate. However, a significant decrease in hardness, particularly in the C-MP10 variant, may lead to excessive brittleness and reduced mechanical integrity, potentially compromising the product’s stability during transport and storage. Therefore, it is important to optimize the level of the MP additive to achieve a balance between desirable brittleness and adequate structural strength.

### 2.3. SDS-Polyacrylamide Gel Electrophoresis Protein Profile

Modifying the composition of snacks, such as cookies and crackers, affects the profile of bioactive ingredients that contribute to their health-promoting properties [22]. Protein molecular weight is crucial in determining the functional, nutritional, and sensory attributes of these snacks. Both high- and low-molecular-weight proteins impact product quality, and specific modifications or hydrolysates can enhance antioxidant activity, texture, and bioavailability. Research suggests that carefully selected recipes can improve the quality and health benefits of snacks like cookies and crackers [23].

**Figure 3:**
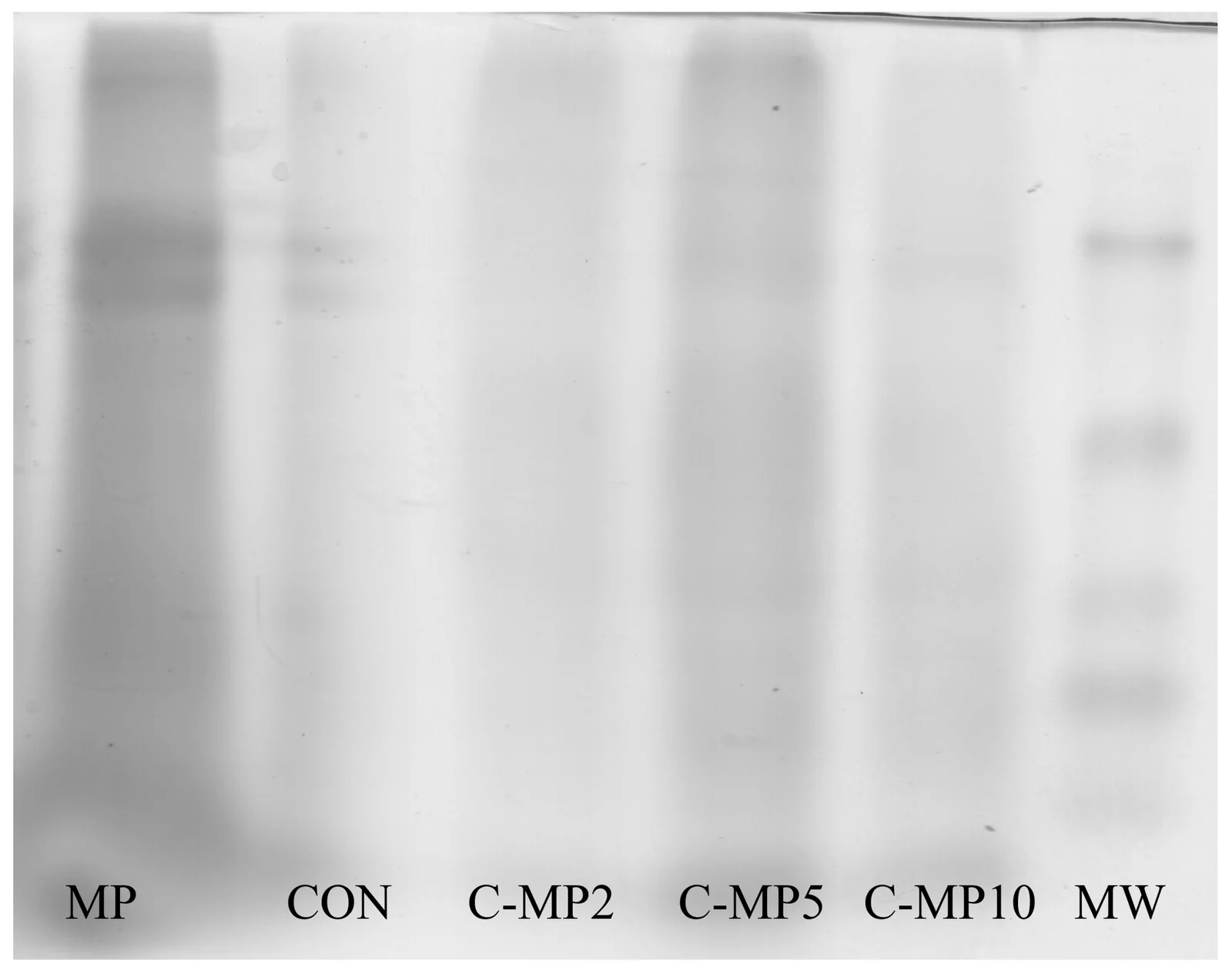
SDS-PAGE protein profile. CON—control sample, C-MP2—sample with 2% mealworm powder addition, C-MP5—sample with 5% mealworm powder addition, C-MP10—sample with 10% mealworm powder addition, MW—molecular weight standard, MP—mealworm powder.

An important aspect of food fortification is the protein profile of the resulting products. To analyze the protein profile, electrophoretic separation of proteins was performed using SDS-PAGE, as illustrated in Figure 3.

Based on the electropherogram, we characterized the protein profile by determining the parameters outlined in Table 2. The following table illustrates clear changes in the protein profile of the crackers as the mealworm powder (MP) content increases. This characterization is based on the separation of proteins by molecular weight using electrophoretic methods, with the proportion of individual bands presented as a volume ratio (%). The control sample (CON) contains five main protein fractions, with molecular weights ranging from approximately 14 to 142 kDa. The largest fraction is the 14.3 kDa protein, making up 35.30% of the total, followed by the 114.4 kDa fraction (22.67%), the 106.9 kDa fraction (16.46%), the 54.4 kDa fraction (13.75%), and the 141.6 kDa fraction (11.82%). This indicates that the protein profile of traditional crackers is predominantly composed of low-molecular-weight proteins, along with selected high-molecular-weight fractions.

Adding 2% mealworm powder significantly altered the protein-fraction distribution. The low-molecular-weight fraction (11.5 kDa) maintained the largest share, increasing from 35.30% to 48.66%. Concurrently, the share of proteins weighing approximately 141 kDa rose notably, from 11.82% to 34.07%. However, the proportions of the intermediate fractions (126.8 kDa, 90.2 kDa, and 65.2 kDa) were relatively small, totaling only about 5–6%. This suggests that a small addition of mealworm powder primarily enriches the product with very high- and very low-molecular-weight proteins, while reducing the relative share of intermediate fractions.

When insect powder was increased to 5%, the protein profile became more complex, with seven distinct protein fractions identified instead of five. The fraction around 10.4 kDa remained dominant, accounting for 47.44% of the total protein. New proteins appeared at approximately 81.8 kDa, 60.2 kDa, and 35.0 kDa, which were not present in the control sample. This increase in protein diversity suggests that a higher proportion of insect powder enhances the variety of proteins in the product. Additionally, the proportion of high-molecular-weight fractions (ranging from 126 to 143 kDa) decreased compared with the sample labelled C-MP2.

The most significant changes were observed after adding 10% mealworm powder. In this case, the fraction weighing approximately 13.7 kDa dominated the profile, accounting for 60.05% of the total protein content. In contrast, the percentage of very high-molecular-weight proteins (140.7 kDa) dropped to just 0.77%, and the fraction weighing 81.6 kDa accounted for only 0.84%. The other proteins (111.6 kDa, 61.3 kDa, 48.0 kDa, and 39.8 kDa) were present at levels ranging from approximately 6% to 17%.

This suggests a shift in the protein profile towards lower-molecular-weight proteins with increased insect powder addition. Notably, the protein profile of mealworm powder differs from that of traditional crackers, in which the highest-molecular-weight proteins (114.98 kDa and 141.43 kDa) are the most abundant. Crucially, the powder itself lacks a predominance of the 10–14 kDa fraction, which is prevalent in all insect powder-enriched crackers. This suggests that proteins undergo structural changes during dough preparation and baking, such as partial hydrolysis, denaturation, or formation of lower-molecular-weight products. However, definitive confirmation of this suggestion requires further analysis.

### 2.4. Nutritional Value

Analysis of the chemical composition of the tested samples indicates statistically significant differences (*p* ≤ 0.05) (Table 3). Changes in the composition of products C-MP2, C-MP5, and C-MP10 relative to the control sample (CON) are clearly correlated with the increasing addition of mealworm powder, which is characterized by a high protein content (54.26 g/100 g) and fat content (31.74 g/100 g).

The protein content increased significantly with the level of MP addition—from 13.10 g/100 g in the control sample to 15.36 g/100 g in the C-MP10 sample. This trend aligns with the high protein concentration in mealworm powder and indicates its effectiveness as an ingredient that enhances the product’s nutritional value. Even with a 10% addition, the increase was over 17% compared to the control sample. From a food technology and nutrition perspective, this increase is significant, as consumers increasingly demand high-protein products.

The fat content increased significantly from 14.56 g/100 g (CON) to 23.42 g/100 g (C-MP10). Similarly, energy increased from 452 kcal/100 g to 491 kcal/100 g. This increase directly from the MP’s high fat content and confirms a strong correlation between lipid content and the product’s caloric value (R = 0.9935, *p* < 0.05). Increasing the energy value may benefit functional products with higher energy density (e.g., for athletes), but it may limit appeal to consumers seeking low-calorie foods.

The ash content increased from 1.33 g/100 g to approximately 1.7 g/100 g in the enriched samples. This indicates that adding MP increased mineral content. The lack of significant differences among the C-MP2, C-MP5, and C-MP10 samples suggests that even a small addition of the raw material significantly affects this parameter; further increases in MP content did not yield a significant increase in ash content, although a slight, statistically insignificant trend was observed. Adding mealworm powder at levels above 10% is likely to significantly increase mineral content.

As the MP addition increased, a significant decrease in carbohydrate content was observed—from 67.26 g/100 g (CON) to 54.62 g/100 g (C-MP10). This is due to the increase in protein and fat content, and MP contains minimal carbohydrates (2.35 g/100 g). From a technological standpoint, reducing carbohydrate content may affect product texture and sensory properties (e.g., crispness, taste).

Moisture content increased in samples with higher MP additions (C-MP5 and C-MP10), reaching values significantly higher than those of the control sample. This may result from the hygroscopic properties of the MP ingredient and its ability to bind water [24]. Increased moisture content may affect product shelf life and texture, potentially shortening the shelf life or requiring modifications to storage conditions.

The results clearly indicate that adding MP significantly enhances the product’s nutritional value by increasing its protein and mineral content. Additionally, the increase in fat content and energy value is an important consideration when designing the final product, especially given consumer expectations. These findings align closely with results from other studies on bread [19], pasta [7], or biscuits [10] enriched with insect powders.

The changes in macronutrient composition show a clear shift in the product’s nutritional profile. It has transitioned from a high-carbohydrate product to a more balanced one, with increased protein and fat content. This transformation could be advantageous given current dietary trends, such as high-protein or low-carbohydrate diets.

### 2.5. Mineral Composition

Edible insects are recognized for their high mineral content. Many species are rich sources of iron, calcium, magnesium, zinc, and copper [25]. As anticipated, the mineral content analysis showed significant differences between the control sample (CON) and the crackers enriched with mealworm powder (Table 4). These changes in mineral concentrations were closely linked to the high mineral content of mealworm powder (MP). As the proportion of MP increased, a consistent rise in the levels of most analyzed elements was noted, confirming its effectiveness as a carrier for the studied mineral components.

Magnesium content increased significantly from 19.47 mg/100 g in the control (CON) to 33.80 mg/100 g in C-MP10. A notable increase was already observed with just a 5% addition of mealworm powder (MP), which has a high magnesium content of 232.75 mg/100 g. Magnesium plays a crucial physiological role, including functions in the muscles and nervous system, so its increased presence greatly enhances the product’s health benefits [26]. Potassium content also increased clearly and significantly, rising from 142.54 mg/100 g to 196.07 mg/100 g. Again, a significant difference was noted with the 5% addition of MP, which is characterized by a high potassium content of 865 mg/100 g. The iron content increased from 1.55 mg/100 g to 2.05 mg/100 g, with even a 5% addition of MP contributing to this increase. This is likely due to MP’s relatively high iron content (3.74 mg/100 g). This increase is nutritionally significant because iron is a key component in preventing anemia [27]. Zinc showed one of the most substantial changes, rising from 0.74 mg/100 g in the control cracker to 1.83 mg/100 g in the C-MP10. The very high zinc content in mealworm powder, at 14.68 mg/100 g, highlights its potential as an ingredient for enriching products with zinc, which plays an important role in immune function and enzymatic processes [28]. The copper content in the CON, C-MP2, and C-MP5 samples remained relatively stable, ranging from approximately 0.14 to 0.15 mg per 100 g. In contrast, the C-MP10 sample showed a significant increase in copper, measuring 0.25 mg per 100 g. Calcium content increased with MP addition, rising from 39.70 mg per 100 g in the CON sample to 46.55 mg per 100 g in the C-MP10 sample. However, these differences were not statistically significant. This indicates that, despite the relatively high calcium content of MP (75.50 mg per 100 g), its contribution at the tested addition levels was insufficient to yield a noticeable fortification effect. The primary source of sodium in the crackers was the salt added per recipe, resulting in consistent sodium levels (~465 to 468 mg per 100 g) across all samples, with no statistically significant differences.

The results show that adding MP improves the product’s mineral profile, particularly magnesium, potassium, iron, and zinc. The increased levels of these minerals confirm that MP is a concentrated source and can be effectively used in the development of functional foods. Notably, the most significant changes occurred at the highest MP inclusion level (10%). This suggests that a minimum inclusion level may be necessary to achieve a nutritionally significant effect. However, from a technological perspective, a trade-off must be considered between enhancing mineral content and the potential changes in sensory and textural characteristics, which—as previously indicated—also undergo significant alterations.

### 2.6. Antioxidant Properties

The antioxidant activity of the analyzed samples, measured as the ability to neutralize ABTS^+^· and DPPH·, as well as the total phenolic compound content (TPC) (mg GAE/100 g), showed significant differences among the tested cracker variants (Table 5). In the ABTS^+^· assay, antioxidant activity increased gradually with increasing MP addition to the crackers. The lowest value was recorded for the control sample (CON: 0.173 ± 0.03 mM TEAC/g sample), while the highest was for C-MP10 (0.469 ± 0.07 mM TEAC/g sample). The CON and C-MP2 samples did not differ significantly, while further increasing MP addition significantly increased in activity (*p* < 0.05). A similar relationship was observed in the DPPH· method, where values increased from 0.109 ± 0.01 mM TEAC/g sample (CON) to 0.32 ± 0.09 mM TEAC/g sample (C-MP10), with statistically significant differences analogous to those observed in the ABTS assay. The phenolic compound content increased in proportion to the MP additive level—from 35.82 ± 0.69 mg GAE/100 g in the control sample to 110.57 ± 1.41 mg GAE/100 g in C-MP10, and all values differed significantly (*p* < 0.05). Pearson’s correlation analysis revealed a very strong positive relationship between phenolic compound content and antioxidant activity. The correlation coefficient between ABTS^+^· and TPC was R = 0.9818 (*p* = 0.018), while an even stronger relationship was observed for DPPH· and TPC (R = 0.9926; *p* = 0.007), indicating that both relationships were statistically significant.

The results clearly indicate that mealworm powder extract is a rich source of polyphenols (418.7 mg GAE/100 g) and that increasing its addition to crackers significantly increases the antioxidant activity of the tested samples. Strong positive correlations between phenolic compound content and antioxidant activity (R > 0.98) confirm the key role of TPC as an indicator of antioxidant potential.

These results align with findings from other studies on food product enrichment using insects. For instance, the antioxidant properties of shortbread cookies, muffins, and pasta improved as the proportion of mealworm powder in the recipe increased [5,10,17]. Different types of insects are excellent sources of antioxidants. Research has shown that adding cricket (*Acheta domesticus*) powder and *Alphitobius diaperinus* powder to bread and sponge cake recipes boosts both the polyphenol content and the antioxidant activity [29,30].

The literature clearly shows that edible insects are a rich source of bioactive compounds, including polyphenols, bioactive peptides, chitin, and other metabolites with biological properties. These compounds exhibit a wide range of activities, such as antioxidant, anti-inflammatory, anticancer, and cardioprotective effects. Edible insects are a source of bioactive compounds that typically originate from plants and accumulate in the insects based on their larval diet. Experimental studies have confirmed the presence of phenolic compounds in insects, including ferulic acid and *p*-coumaric acid, and have demonstrated their significant antioxidant activity in ABTS and DPPH assays [31].

The findings outlined are significant in the field of public health, as oxidative stress is a key factor in the development of various lifestyle-related diseases, including cardiovascular disease, type 2 diabetes, cancer, and neurodegenerative disorders. The increased DPPH and ABTS radical-scavenging activity demonstrates enhanced in vitro antioxidant capacity of the MP-enriched crackers. However, these chemical assays do not directly predict antioxidant effects in vivo, and the physiological relevance of these findings requires further investigation. However, research indicates that bioactive compounds in insects may help regulate inflammatory processes and lipid and glucose metabolism, as well as support cardiovascular health [32].

### 2.7. LC-MS/MS Analysis of Phenolic Compounds

#### 2.7.1. Comparison of TICs

**Figure 4:**
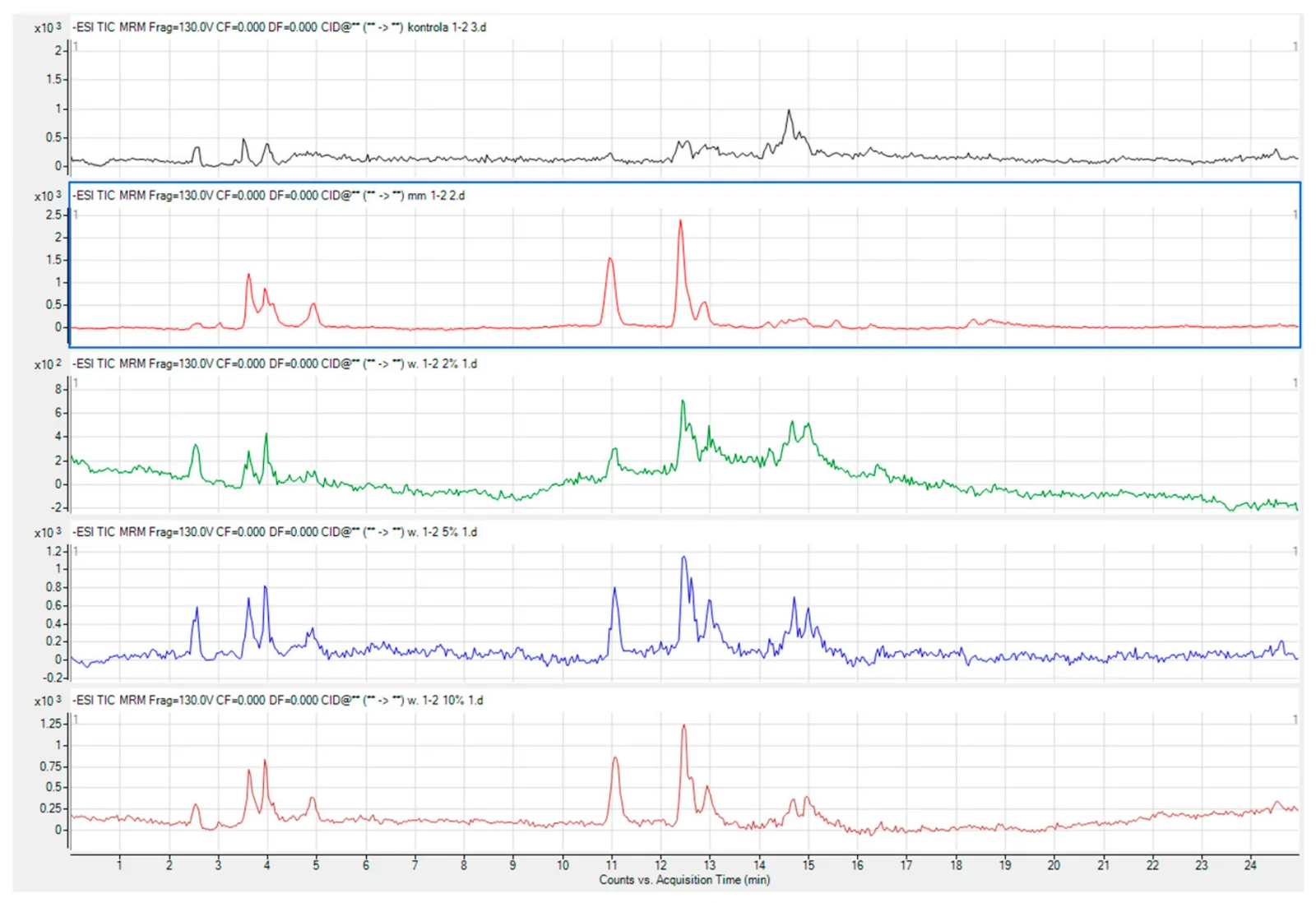
Total ion chromatograms (TICs) obtained for the control sample, *Tenebrio molitor* powder, and products enriched with 2%, 5%, and 10% insect powder under identical LC-MS/MS conditions.

Total ion chromatograms (TICs) obtained for the control sample, *Tenebrio molitor* powder, and products enriched with 2%, 5% and 10% insect powder revealed noticeable differences in their overall chemical profiles (Figure 4). The chromatogram of *T. molitor* powder was characterized by several distinct signals, particularly within the retention time of approximately 3–5 min and 11–13 min, which were absent or considerably less pronounced in the control sample. These observations indicate differences in the composition and relative abundance of compounds present in the analyzed materials.

The chromatographic profiles of the enriched products exhibited features intermediate between those of the control sample and insect flour. Several peaks characteristic of *T. molitor* powder were also observed in the enriched samples, suggesting the contribution of insect-derived compounds into the cereal matrix. In particular, signals detected in the retention time region between 11 and 13 min became progressively more pronounced in samples containing 2%, 5%, and 10% insect flour.

The appearance of these common chromatographic features suggests that compounds originating from *T. molitor* powder contribute to the chemical composition of the enriched products. The observed differences in TIC profiles provided preliminary evidence of qualitative variation among the analyzed samples and justified further investigation using a targeted suspect-MRM screening approach to identify of specific phenolic compounds. Among the enriched formulations, C-MP10 was selected for subsequent detailed qualitative suspect-MRM analysis because the highest level of *T. molitor* powder supplementation generally provided more pronounced signals for the monitored phenolic compounds and thus offered the greatest likelihood of detecting compounds associated with the insect-derived ingredient.

#### 2.7.2. Qualitative Identification of Phenolic Compounds

Suspect-MRM screening of the targeted phenolic compounds enabled the detection of 13 analytes in the investigated samples. The identities of these compounds were confirmed by comparison of their retention times and characteristic MRM transitions with those obtained for authentic reference standards analyzed under identical chromatographic conditions. Since no analytical standard was available for 4-hydroxyacetophenone, the corresponding signal was tentatively assigned solely on its characteristic MRM transition. The confirmed compounds together with tentatively assigned 4-hydroxyacetophenone, their retention times where applicable, and qualitative assessment of signal intensities in the analyzed samples are summarized in Table 6. The list of monitored compounds together with their corresponding MRM transitions is presented in Supplementary Table S1.

As shown in Table 6, the qualitative phenolic profiles differed among the analyzed samples. The highest number of detected compounds and the strongest signal intensities were generally observed in *T. molitor* powder, indicating that insect powder contributed distinct phenolic constituents to the analyzed material.

The distribution of individual phenolic compounds among the analyzed samples suggests differences in their origin and contribution to the overall phenolic profile. Ferulic acid exhibited the highest signal intensity in the control sample, which is consistent with previous reports identifying this compound as one of the predominant phenolic acids in wheat grain and wheat-derived products. Similarly, rutin and apigenin-7-O-glucoside were detected primarily in the control sample, indicating their association with the cereal matrix [33,34].

In contrast, protocatechuic acid, caffeic acid, protocatechuic acid aldehyde were detected at higher signal intensities in *T. molitor* powder than in the control sample. Furthermore, 4-hydroxybenzoic acid and the signal tentatively assigned to 4-hydroxyacetophenone were absent in the control sample but present in both *T. molitor* powder and the enriched product. These findings suggest a possible association of these signals that these compounds may be associated with the insect-derived material and contribute to the phenolic composition of the fortified formulation [35].

Interestingly, luteolin was detected exclusively in *T. molitor* powder, whereas chlorogenic acid was observed only at trace levels in both the control sample and insect flour and was not detected in the enriched product. The absence of some compounds in the enriched product despite their presence in insect flour may be related to their relatively low abundance and matrix-related effects influencing signal detection; however, this requires more extensive research.

Overall, supplementation with *T. molitor* powder modified the qualitative phenolic profile of the enriched product with several phenolic compounds or characteristic signals being absent or detected only at trace levels in the control sample. These findings suggest that insect flour may serve as an additional source of phenolic constituents and contribute to the overall phenolic composition of the final formulation. However, these findings are preliminary, and further quantitative and confirmatory studies are needed to fully evaluate the contribution and potential significance of detected phenolic constituents.

### 2.8. Starch Digestibility and Predicted Glycemic Index

The rate at which glucose is released from starch during digestion is the primary determinant of the glycemic index of starchy foods. Numerous studies have shown that this rate can be predicted using in vitro starch digestibility tests [36]. Rapidly digestible starch (RDS) is absorbed quickly, while slowly digestible starch (SDS) is released more gradually. Although SDS provides energy in the form of carbohydrates, it does not lead to a significant glycemic response in people without glucose intolerance [37].

The levels of rapidly digestible starch (RDS), slowly digestible starch (SDS), and the predicted glycemic index (pGI) exhibited significant variation among the samples analyzed (Table 7). For RDS, a clear decrease was noted as the amount of mealworm powder increased. The control sample had the highest RDS content (139.48 ± 3.16 mg/g), while sample C-MP10 had the lowest (93.44 ± 3.81 mg/g). Differences across all mealworm powder addition levels were statistically significant, although samples C-MP2 and C-MP5 did not differ significantly from each other (*p* < 0.05). In contrast, SDS content increased with mealworm powder addition, rising from 101.28 ± 1.35 mg/g in the control sample to 132.31 ± 2.25 mg/g in C-MP10. All analyzed samples exhibited statistically significant differences, demonstrating a consistent effect of mealworm powder addition on the starch fraction characteristics (*p* < 0.05). The predicted glycemic index (pGI) values also decreased slightly but significantly as mealworm powder content increased. The control sample (56.32 ± 0.35) and C-MP2 (55.84 ± 0.1) had the highest pGI values and did not differ significantly. However, significantly lower values were observed in samples C-MP5 (54.94 ± 0.07) and C-MP10 (54.82 ± 0.09) (*p* < 0.05). Although C-MP10 showed a statistically significant reduction in pGI compared with the control (54.82 vs. 56.32), the absolute difference was modest (1.50 pGI units). Therefore, the observed effect should be interpreted primarily as evidence that cricket powder slightly modified in vitro starch digestibility, rather than as evidence of a physiologically relevant reduction in postprandial glycemic response.

The results indicate that adding MP significantly impacts starch digestibility by reducing the proportion of rapidly digestible starch (RDS) and increasing the proportion of slowly digestible starch (SDS). This change is particularly important from a nutritional standpoint; a high RDS content is linked to a rapid release of glucose into the bloodstream, while SDS contributes to a more gradual glucose release, helping to stabilize blood sugar levels [37]. By utilizing the glycemic index (GI), we can measure the relative increase in blood glucose levels after consuming a carbohydrate-rich meal [38]. The metabolic and glycemic response to food products can be estimated using the in vitro starch hydrolysis method [39]. We can alter the glycemic index of foods in various ways, such as through appropriate cooking and processing methods, and by modifying product recipes to enhance their ingredients that influence starch digestibility [5,40]. Although the differences in pGI values are relatively small, they remain statistically significant. The present study did not directly investigate the mechanisms underlying these changes. Nevertheless, the modified macronutrient composition of the enriched crackers provides several plausible explanations. This improvement may result from interactions between MP components and the starch matrix that limit enzymatic starch digestion, thereby slowing hydrolysis. These findings are consistent with the existing literature, which suggests that the presence of proteins, dietary fiber, and phenolic compounds can reduce starch digestibility by forming complexes or physically obstructing access to digestive enzymes [41,42]. Given previous research indicating high protein, lipid, and phenolic content, as well as antioxidant activity, we can infer that proteins and bioactive compounds significantly influence digestive properties in this context. These effects can occur in several ways. One example is the interaction between starch, lipids, and proteins through electrostatic and hydrophobic forces, which results in improved structural organization and reduced digestibility [41]. Additionally, phenolic compounds can inhibit amylolytic enzyme activity, thereby delaying glucose absorption [42]. Another potential mechanism involves the formation of viscous protein-fiber networks, which can trap starch granules and limit their availability to digestive enzymes [43]. The present study did not specifically examine the mechanisms underlying these observations and should therefore be regarded as possible explanations rather than demonstrated causal relationships.

### 2.9. Consumer Acceptance Analysis

Consumer acceptance is crucial in selecting specific food products. Therefore, when developing new recipes, it is essential to conduct organoleptic evaluations with potential consumers as a necessary part of product development research. Table 8 presents the results of the consumer acceptance evaluation of the proposed crackers, conducted using a five-point hedonic scale.

The sensory analysis revealed significant differences between the control sample and the mealworm powder-enriched products, with the nature of these changes varying by the quality attribute evaluated. The results indicate that adding mealworm powder affects perceptions of visual and taste characteristics, as well as the product’s overall acceptability. The strongest effect was observed for color (χ^2^(3) = 57.112, *p* < 0.001, Kendall’s W = 0.307). Mean color scores decreased progressively with increasing insect powder addition level. The control sample received the highest rating of 4.33, while the samples with higher levels of mealworm powder showed a systematic decrease in visual acceptability, dropping to 2.73 for sample C-MP10. This suggests that a higher concentration of mealworm powder significantly affects color perception, making the product less visually appealing.

Crispness showed a different trend, with ratings remaining relatively stable within a narrow range of 3.71 to 4.05. The lack of statistically significant differences (Friedman test, *p* = 0.189) suggests that, despite changes in instrumental measurements of hardness and fracture time, the panelists’ perception of crispness remained consistent. This may indicate that any structural differences did not exceed the threshold of sensory perception or were compensated for by other factors, such as the sound produced during biting or the product’s pore structure. These findings also highlight that instrumental fracture parameters and sensory crispness represent related but distinct aspects of cracker texture.

In terms of smell, ratings differed significantly among formulations (χ^2^(3) = 18.097, *p* < 0.001, W = 0.097). The 2% and 10% formulations received significantly higher aroma scores than the control sample, whereas the 5% formulation did not differ significantly from the control. Interestingly, insect powder incorporation did not adversely affect aroma perception. In fact, the 2% and 10% formulations received significantly higher aroma scores than the control, while the 5% formulation remained comparable to the control. This finding suggests that the characteristic aroma introduced by insect powder was not perceived negatively under the conditions of the present study. However, because the effect size was small (Kendall’s W = 0.097), the differences should be interpreted cautiously. Moreover, the absence of a clear dose-dependent response indicates that aroma perception was not directly related to increasing insect powder concentration.

The taste score was highest for the control sample and the sample with 2% MP, at 3.72 and 3.85, respectively. However, as enrichment increased, taste scores gradually decreased, although the differences were not statistically significant. Nevertheless, the small effect size (Kendall’s W = 0.083) indicates that these differences should be interpreted cautiously.

The overall evaluation showed that the control sample and C-MP2 achieved the highest acceptance ratings of 4.02 and 4.29, respectively. In contrast, further increases in MP addition led to a significant decline in product acceptance, with scores dropping to 3.60 for C-MP5 and 3.10 for C-MP10. All pairwise comparisons between formulations were significant after Holm correction (*p* ≤ 0.026). These findings indicate that a small MP addition enhances the product’s overall appeal, while higher levels adversely affect sensory quality.

The ranking method results align with those obtained from the five-point scale. The sample with a 2% addition of mealworm powder received the highest rating, with a score of 132 points. The control sample followed with 110 points. Conversely, the sample with the highest enrichment of mealworm powder received the lowest ratings, totaling only 62 points.

Accepting insects as food is complex, and food neophobia often plays a significant role in consumer attitudes. This makes the findings particularly important, as they demonstrate that crackers enriched with a small amount of insects received more favorable reviews than the control sample. A comprehensive review by Alhujaili et al. [12] suggests that consumers are more likely to be drawn to products containing processed insects in convenient forms. To increase acceptance, it may be beneficial to develop products that consumers recognize as familiar in terms of both the food base and taste—such as cakes, muffins, and pasta. This approach can help reduce product novelty, thereby decreasing food neophobia.

### 2.10. FTIR

**Figure 5:**
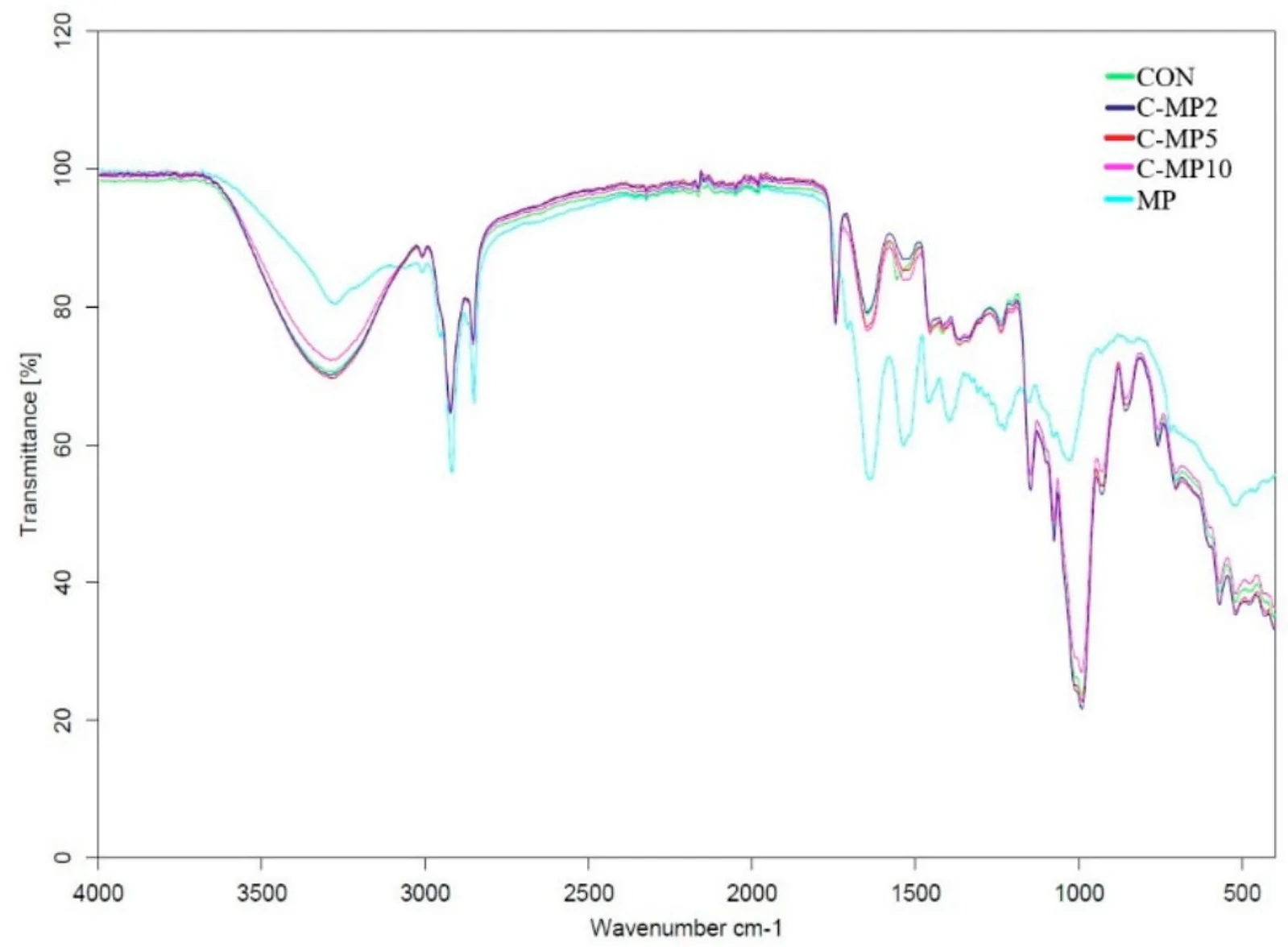
FT-IR spectra of the studied crackers and mealworm powder. CON—control sample, C-MP2—sample with 2% mealworm powder addition, C-MP5—sample with 5% mealworm powder addition, C-MP10—sample with 10% mealworm powder addition.

The FTIR spectra of the control and mealworm-powder-enriched crackers showed broadly similar spectral profiles, whereas the spectrum of mealworm powder exhibited differences in the relative intensity of several absorption regions (Figure 5). The cracker spectra showed bands in regions commonly associated with proteins, lipids, and carbohydrates. Changes were observed in the region around1650 cm^−1^ (Amide I) and ~1540 cm^−1^ (Amide II), along with a band around 3300 cm^−1^ associated with the vibrations of –OH and –NH groups. These spectral changes align with compositional data showing increases in protein (from 13.10% to 15.36%) and fat (from 14.56% to 23.42%). Thus, some differences between the FTIR profiles of the formulations may reflect their different chemical composition after incorporating mealworm powder [44]. Differences were also visible in the carbohydrate-dominated fingerprint region, particularly around 1000–1100 cm^−1^, where C–O and C–O–C vibrations of carbohydrate structures contribute to the spectrum. As wheat flour was progressively replaced by mealworm powder, carbohydrate content decreased from 67.26% in CON to 54.62% in C-MP10. Consequently, changes in this spectral region may reflect both the reduced contribution of cereal carbohydrates and alterations in their local molecular environment within the multicomponent cracker matrix.

Interestingly, the FTIR spectral differences occurred concurrently with independently measured changes in starch digestibility and texture. RDS decreased from 139.48 mg/g in CON to 93.44 mg/g in C-MP10, whereas SDS increased from 101.28 to 132.31 mg/g and pGI decreased from 56.32 to 54.82. These findings demonstrate that mealworm powder incorporation modified starch digestion behavior; however, the present FTIR data do not establish the molecular mechanism responsible for these changes. The higher protein and lipid contents of the enriched formulations and their possible interactions with starch provide plausible explanations, as protein–starch and starch–lipid interactions have previously been reported to influence starch accessibility and digestion. Proteins can create a physical barrier that limits access for amylolytic enzymes [45], while lipids facilitate the formation of amylose–lipid complexes, which are less susceptible to enzymatic hydrolysis [46]. These mechanisms should therefore be regarded as hypotheses requiring further structural confirmation. Likewise, the reduction in cracker hardness and fracture time indicates changes in the product’s mechanical properties but cannot be directly attributed to the FTIR spectral differences. Partial replacement of wheat flour by mealworm powder altered the relative proportions of carbohydrates, proteins, and lipids and could have affected the continuity of the cereal matrix. Therefore, the FTIR results should be considered complementary evidence of compositional and local molecular-environment differences among the formulations rather than direct evidence for a specific mechanism determining cracker texture.

## 3. Materials and Methods

### 3.1. Materials

Wheat flour containing 10.6% protein, 1.3% fat, and 72% carbohydrates were supplied by GoodMills Polska sp. z o.o., Grodzisk Wielkopolski, Poland. The remaining food ingredients included salt (Cenos sp. z o.o., Września, Poland), sugar (Pfeifer & Langen Polska S.A., Środa Wielkopolska, Poland), rapeseed oil (Bunge Polska Sp. z o.o., Kruszwica, Poland), eggs (Oldar Group Sp. z o.o., Komorów, Poland) and baking powder (FoodCare Sp. z o.o., Zabierzów, Poland). All of these ingredients were obtained from a retail store in Lublin, Poland. Mealworm larvae (*Tenebrio molitor* L., Coleoptera: Tenebrionidae) were sourced from Bugstore (Kraków, Poland).

Analytical reagents, including Trolox (6-hydroxy-2,5,7,8-tetramethylchroman-2-carboxylic acid), ABTS (2,2′-azinobis(3-ethylbenzothiazoline-6-sulfonic acid), DPPH (2,2-diphenyl-1-picrylhydrazyl), dinitrosalicylic acid, pepsin from porcine gastric mucosa (10,080, 250 U/mg), pancreatin from porcine pancreas (P1750, 8× USP specifications), amyloglucosidase (10,115, 70 U/mg), and invertase (I4504, 300 U/mg) were purchased from Sigma-Aldrich Company, Ltd. (St Louis, MO, USA). The D-glucose oxidase/peroxidase assay kit (GOPOD format) was supplied by Megazyme International (Wicklow, Ireland). Unless otherwise specified, all chemicals and reagents were of analytical grade.

### 3.2. Production of Insect Powder

Mealworms underwent a 48 h fasting period before processing to clear gastrointestinal contents. The insects were then frozen at −18 °C for 24 h, followed by lyophilization using a Delta 2–24 LSCplus freeze dryer (Christ, Osterode am Harz, Germany). Lyophilization was carried out for 48 h at a pressure of 0.521 mbar, with the shelf and chamber temperatures maintained at 20 °C and −65 °C, respectively. The dried insects were then milled and sieved through a 20-mesh screen to obtain a homogeneous powder. The prepared mealworm powder was vacuum-sealed and maintained under frozen storage conditions until further use.

### 3.3. Production of Crackers

The crackers were prepared in three variants with different amounts of mealworm powder added (MP), partially replacing wheat flour at 2% (C-MP2), 5% (C-MP5), and 10% (C-MP10) by weight. The control cracker (CON) contained no insect powder.

The reference cracker consisted of wheat flour, egg, sugar, baking powder, salt, rapeseed oil and water. Table 9 shows the detailed composition of the control and supplemented crackers. The ingredients were mixed, and the dough was kneaded on a pastry board. The prepared dough was rolled out to about 5 millimeters thick and cut into equal-sized squares. The crackers were baked at 180 °C for 15 min. The crackers were produced in three independent batches.

### 3.4. Colour Measurements

Color measurements were conducted using an EnviSense colourimeter NH310 (nh3, China). The CIE L*a*b* scale recorded color differences in lightness (L*) and color (a*—redness, b*—yellowness). Color parameters of the cracker samples were measured in five repetitions for each sample batch. The total color difference (∆E) was calculated using the formula below:

(1) $$∆E=\;\sqrt{∆L^{2+}{∆a}^{2}+{∆b}^{2}},$$

where

where ΔL*, Δa*, and Δb* are differences in the L*, a*, and b* values between the reference sample and the test sample, respectively.

The browning index was calculated as follows:

(2) $$\mathrm{BI}=\;\frac{100(x-0.31)}{0.17},$$

where x:

(3) $$x=\frac{\left(a^{*}+1.75L^{*}\right)}{\left(5.645L^{*}\;+\;a^{*}-3.012b^{*}\right)},$$

### 3.5. Texture Parameters

The texture parameters of the obtained crackers were measured 1 day after baking, using the method described by Djouadi et al. [16] with slight modifications. An AMETEK Brookfield CTX texture analyser (Canada) was used to perform a penetration test (5 mm depth) on a perforated base using a 2 mm diameter stainless steel probe at 3 mm/s and a 15 kg load cell. During the test, hardness and fracture time were determined. Hardness was expressed as the maximum force required to fracture the sample, whereas fracture time represented the time elapsed until the first major fracture event under the applied test conditions [47]. Texture analysis of the cracker samples was carried out in five repetitions for each sample batch.

### 3.6. Protein Isolation

The mealworm and cracker protein were isolated by alkaline extraction. Mealworm powder and crackers were mixed with 0.2% NaOH (1:10, *w*/*v*). The samples were shaken at room temperature for 1 h, then centrifuged at 8000× *g* for 30 min. The protein from the supernatant was precipitated at pI (4.3) and centrifuged at 8000× *g* for 30 min. The protein isolate was stored at 4 °C for SDS-PAGE analysis.

### 3.7. SDS-PAGE Electrophoresis

SDS-PAGE analysis of *protein* samples was performed on 5% concentrated and 12% separated polyacrylamide gels. A Mini-Protean BioRad electrophoresis system (BioRad, Hercules, CA, USA) with 20 mA was used. The samples were mixed with buffer samples (BioRad, 1:1, *v*/*v*) and heated at 100 °C for 5 min prior to electrophoresis. The gels were stained with Coomassie Blue R-250 using a molecular marker ranging from 20 to 120.0 kDa (Molecular Weight Marker #SM0441, Fermentas) as a standard.

### 3.8. Nutritional Value

Moisture, ash, fat, and protein content were determined using standard analytical methods [48]. The carbohydrate content was calculated as 100% minus the sum of ash, fat, protein, and moisture. The energy value was calculated using a conversion method [49].

### 3.9. Mineral Content

Using flame atomic absorption spectrophotometry (FAAS, Solaar 939, Unicam), the concentration of the most common minerals—Ca, Fe, K, Mg, Na, Cu and Zn—in cracker mineralisation was determined, following the method described by Jorhem and Engman [50]. 0.5 g of each cooked and lyophilized cracker sample was mixed with 4 mL of nitric acid (V). Then, the samples in closed vessels were mineralized in a microwave oven (Mars, Xpress, CEM Corporation, Matthews, NC, USA).

### 3.10. Antioxidant Properties

#### 3.10.1. Extraction of Bioactive Compounds

A 1 g sample of lyophilised and ground crackers was extracted in a laboratory shaker for 120 min using 10 mL of a 4:1 ethanol/water (*v*/*v*) solution. The mixture was then centrifuged at 3000 g for 10 min, and the resulting supernatant was stored at –20 °C for subsequent analysis [51].

#### 3.10.2. DPPH Radical Scavenging Activity

The DPPH· scavenging capacity was determined using a slightly modified method based on that described by Brand-Williams et al. [52]. In brief, 0.1 mL of the sample was mixed with 2.9 mL of a 6 µM DPPH· solution prepared in 75% methanol. After allowing the reaction to proceed for 30 min, the absorbance was recorded at 515 nm.

(4) Scavenging activity (%) = [1 − (A sample/A control)] × 100,

where A sample is the absorbance of the mixture of sample and DPPH·; A control is the absorbance of the control (DPPH· solution).

The results were expressed as Trolox Equivalent Antioxidant Activity (TEAC) values (mM Trolox/g sample).

#### 3.10.3. ABTS Radical Scavenging Activity

The ABTS^+^· scavenging activity was determined using a modified procedure based on the method described by Re et al. [53]. Specifically, 0.1 mL of the sample was combined with 2.9 mL of an ABTS^+^· solution. After a 30-minute reaction, the absorbance was measured at 734 nm.

(5) Scavenging activity (%) = [1 − (A sample/A control)] × 100,

where A sample is the absorbance of the mixture of sample and ABTS^+^·; A control is the absorbance of the control (ABTS^+^· solution).

The results were expressed as Trolox Equivalent Antioxidant Activity (TEAC) values (mM Trolox/g sample).

#### 3.10.4. Total Phenolic Content

The total phenolic content (TPC) of the samples was determined using the Folin–Ciocalteu spectrophotometric method, as described by Singleton and Rossi [54]. A 0.04 mL aliquot of the extract was mixed with 3.16 mL of distilled water and 0.2 mL of Folin–Ciocalteu reagent. After vigorous vortex mixing, 0.6 mL of saturated sodium carbonate was added. The mixture was then incubated at 40 °C for 30 min. Absorbance was measured at 725 nm against a blank, and the results were expressed as milligrams of gallic acid equivalents (GAE) per 100 gram of sample.

#### 3.10.5. LC-MS/MS Analysis of Phenolic Compounds

Phenolic compounds were analyzed using an Agilent 1290 Infinity liquid chromatography system coupled to an Agilent 6460 Triple Quadrupole LC/MS mass spectrometer (Agilent Technologies, Santa Clara, CA, USA) equipped with an electrospray ionization (ESI) source operating in negative ionization mode. Chromatographic separation was performed on a Zorbax Eclipse Plus-C18 column (4.6 × 100 mm, 3.5 µm, Agilent Technologies, USA). The column oven was set at 40 °C. The mobile phase consisted of 0.1% acetic acid in water (A) and 0.1% acetic acid in acetonitrile (B) delivered at a flow rate of 0.3 mL min^−1^. The gradient program was as follows: 0–5 min, 5% B; 5–10 min, 5–30% B; 10–20 min, 30–35% B; 20–23 min, 35–95% B; 23–25 min, 95–5% B; followed by re-equilibration to the initial conditions. The injection volume was 2 µL. All extracts were diluted 1:1 and filtered through 0.22 µm syringe filters before injection into the LC-MS/MS system.

The ESI source parameters were set as follows: gas temperature, 330 °C; gas flow, 12 L min^−1^; nebulizer pressure, 40 psi; sheath gas temperature, 350 °C; sheath gas flow, 11 L min^−1^; and capillary voltage, 4500 V.

Qualitative identification of phenolic compounds was performed using a suspect-MRM strategy. A total of 25 targeted phenolic compounds, including phenolic acids, phenolic aldehydes, and flavonoids, were selected based on previously published studies describing phenolic constituents of wheat flour and *Tenebrio molitor* larvae [33,34,35], as well as the availability of analytical standards. The identity of the detected analytes was confirmed by comparison of their retention times and characteristic MRM transitions with those obtained for authentic reference standards analyzed under identical LC-MS/MS conditions. Retention time agreement with the corresponding reference standard within a tolerance of ± 0.3 min, together with the presence of the characteristic MRM transitions, was used as the identification criteria. As the present method was intended for qualitative profiling rather than quantitative determination, the monitored MRM transitions were used for compound identification and were not designated as quantifier and qualifier transitions. Authentic reference standards were available for all confirmed compounds. In the case of 4-hydroxyacetophenone, for which no authentic reference standard was available, the corresponding signal was tentatively assigned based solely on its characteristic MRM transitions. The list of monitored compounds together with their corresponding MRM transitions is presented in Supplementary Table S1.

Blank samples consisting of the extraction solvent were analyzed to monitor potential background signals and carry-over. A mixture of 12 authentic reference standards was analyzed to verify retention times and characteristic MRM transitions. All samples were analyzed in triplicate to assess the reproducibility of the qualitative results. A signal was considered detectable when a distinct and reproducible chromatographic response for the characteristic MRM transition was observed against a stable, low-noise baseline and was absent from the corresponding blank.

### 3.11. In Vitro Digestibility of Starch

In vitro starch digestibility was determined using a slightly modified protocol based on the method described by Monro et al. [39]. Briefly, 1 g of the sample was mixed with 30 mL of water and 0.8 mL of 1 M HCl to adjust the pH to 2.5. Gastric digestion was initiated by adding 1 mL of a 10% pepsin solution prepared in 0.05 M HCl. The mixture was then incubated at 37 °C for 30 min with constant agitation at 130 rpm.

To simulate small-intestinal conditions, 2 mL of 1 M NaHCO_3_ and 5 mL of 0.1 M phosphate buffer (pH 6) were added. Subsequently, 4.6 mg of amyloglucosidase and 5 mL of 2.5% pancreatin, both prepared in 0.1 M phosphate buffer (pH 6), were introduced. The hydrolysate volume was adjusted to 55 mL with distilled water.

Samples (1.0 mL) were taken at 0, 20, 30, 60, 90, 120, and 180 min after the start of enzymatic hydrolysis. Each aliquot was immediately combined with 4 mL of absolute ethanol to stop enzyme activity.

### 3.12. Rapidly and Slowly Digested Starch Contents

The contents of rapidly digestible starch (RDS) and slowly digestible starch (SDS) were quantified by measuring the amount of reducing sugars released during the in vitro digestion of crackers using a modified method based on Soong et al. [55]. The released sugars, expressed as monosaccharides, were determined using the dinitrosalicylic acid (DNS) colorimetric assay.

To achieve complete depolymerisation into monosaccharides, 50 mL of the supernatant was combined with 0.25 mL of acetate buffer containing 0.4% invertase and 1% amyloglucosidase, then incubated at room temperature for 30 min. Next, 0.75 mL of the DNS reagent mixture (consisting of 0.5 mg/mL glucose, 4 M NaOH, and DNS solution in a 1:1:5 ratio) was added, and the samples were heated at 95–100 °C for 15 min. After cooling, 4 mL of distilled water was added to dilute the samples, and the absorbance was measured at 530 nm using a blank as reference. The quantity of released sugars was expressed as milligrams of glucose per gram of sample as well as per gram of dry matter. RDS was defined as the amount of reducing sugars released after 20 min of intestinal digestion, whereas SDS was calculated as the difference between the reducing sugars measured after 120 min and the RDS value.

### 3.13. Predicted Glycemic Index

Predicted glycemic index (pGI) of crackers was determined using the method described by Reis and Abu-Ghannam [56]. Cracker samples were subjected to enzymatic digestion, and 1 mL aliquots were collected at specific time intervals. Enzyme activity was immediately stopped by adding 4 mL of absolute ethanol to each collected aliquot.

Glucose levels were determined using the GOPOD assay and plotted over time. The areas under the hydrolysis curves (AUCs) were calculated, and the hydrolysis index (HI) was expressed as a percentage by comparing the AUC of each sample with that of the reference product (white bread). The HI values were additionally adjusted based on the total available carbohydrate content of both the sample and the reference. The predicted glycemic index was then calculated using the equation proposed by Goñi, Garcia-Alonso, and Saura-Calixto [57].

(6) GI (%) = 39.71 + 0.549 × HI,

### 3.14. Fourier-Transform Infrared (FTIR) Analysis

The infrared spectra of cracker samples were recorded using an FTIR spectrometer (FT-IR ALPHA II, Bruker Optics Inc., Billerica, MA, USA) employing the Attenuated Total Reflectance (ATR) technique and subtracting air background spectra. The spectra were collected over a frequency range of 4000 cm^−1^ to 400 cm^−1^ in a 26-scan interferogram with a resolution of 4 cm^−1^, then smoothed and baseline corrected using Opus software (Opus, Bruker Optics Inc., Billerica, MA, USA).

### 3.15. Consumer Acceptance Analysis

The semi-consumer analysis was conducted by 70 panellists, randomly selected from students at the University of Life Sciences in Lublin, Poland. Before the evaluation, participants were asked whether they had previously consumed insect-containing foods, and previous experience with insect-based foods was used as an exclusion criterion. Prior to the sensory evaluation, all participants were informed that the tested products contained edible insects and signed an informed consent form. The consent form included information on the potential allergenicity of insect-containing products, particularly for individuals with known allergies to crustaceans and house dust mites. For sensory evaluation, each cracker sample was served in approximately 5 g portions at room temperature (approximately 21 °C). Samples were presented on plates and identified using randomly assigned three-digit codes. The order in which the items were arranged on the plate was randomized among participants to minimize potential order effects. Between samples, participants were given room-temperature water as a palate cleanser and instructed to cleanse their palate before evaluating the next sample. The color, aroma, texture, taste, and overall acceptability of the crackers were assessed on a scale of 1 to 5 (5—excellent, 1—extremely unsatisfactory). Evaluation conditions were kept constant.

The second test evaluated consumer preferences. Participants ranked coded cracker samples by acceptability on a scale from 1 (most acceptable) to 4 (least acceptable). To compile the results, point values were assigned to the ranked crackers, enabling us to create an overall ranking of consumer preferences.

### 3.16. Statistical Analysis

For each independently prepared batch, each analytical determination was performed in triplicate as technical replicates, unless stated otherwise, and averaged. The results are reported as mean values ± standard deviation (SD). Statistical analysis was conducted using Statistica software (version 13.0, StatSoft, Kraków, Poland). Differences among group means were assessed by one-way analysis of variance (ANOVA), followed by Tukey’s Honestly Significant Difference (HSD) test for post hoc comparisons, with statistical significance established at *p* < 0.05. The correlations between the selected results from the four treatment means were analyzed using Pearson’s correlation test.

Sensory scores were treated as ordinal data. Because the same panellists evaluated all four cracker formulations, differences among formulations were assessed using the Friedman test for related samples. When the Friedman test indicated a significant overall difference, pairwise comparisons were performed using the Wilcoxon signed-rank test, with *p*-values adjusted using the Holm method to account for multiple comparisons. Descriptive statistics are presented as mean ± standard deviation and median with interquartile range (IQR). Kendall’s coefficient of concordance (W) was calculated as a measure of effect size for the Friedman test. Statistical significance was set at *p* < 0.05.

## 4. Conclusions

Mealworm powder proved to be a promising ingredient for modifying the nutritional and functional characteristics of crackers. Incorporating it at 2–10% increased protein, fat, mineral, and phenolic contents and antioxidant activity while reducing carbohydrate content. Supplementation also modified the protein profile, with low-molecular-weight fractions (10–14 kDa) becoming increasingly predominant. FTIR analysis revealed qualitative differences in spectral regions associated with proteins, lipids, and carbohydrates, consistent with the altered composition of the enriched formulations. However, these spectral differences cannot be considered direct evidence of specific intermolecular interactions or changes in molecular organization. Mealworm powder incorporation was also associated with lower RDS, higher SDS, and a small reduction in pGI, while hardness and fracture time decreased with increasing supplementation. Higher inclusion levels resulted in progressively darker crackers and reduced sensory acceptance, whereas the 2% mealworm-powder formulation achieved the highest overall consumer acceptance. Overall, mealworm powder may be used to enhance selected nutritional and functional characteristics of crackers, although its incorporation level should be optimized to balance nutritional improvement with technological and sensory quality.

## Figures and Tables

**Table 1:** Color parameters (L*, a*, b*), color difference (∆E) and browning index (BI) of crackers.

Sample	L*	a*	b*	ΔE	BI
CON	78.01 ± 1.33 ^a^	6.43 ± 0.55 ^c^	28.53 ± 0.61 ^a^	-	50.91
C-MP2	72.62 ± 1.33 ^b^	7.92 ± 0.54 ^bc^	28.20 ± 1.61 ^a^	5.60	56.45
C-MP5	64.51 ± 1.14 ^c^	10.09 ± 1.80 ^b^	29.91 ± 1.94 ^a^	14.06	72.54
C-MP10	55.60 ± 0.78 ^d^	13.32 ± 0.23 ^a^	30.55 ± 0.49 ^a^	21.02	89.01

Values with different letters within the same column are significantly different (*p* ≤ 0.05). CON—control sample, C-MP2—sample with 2% mealworm powder addition, C-MP5—sample with 5% mealworm powder addition, C-MP10—sample with 10% mealworm powder addition.

**Table 2:** Characteristics of molecular weight distribution peaks identified in studied samples.

Band	Peak Value	Peak Rf	Molecular Weight	Raw Volume	Volume Ration (%)
CON					
1	4	0.057	141.601	367	11.820
2	11	0.239	114.383	704	22.673
3	8	0.289	106.945	511	16.457
4	2	0.640	54.368	427	13.752
5	11	0.908	14.302	1096	35.298
C-MP2					
1	6	0.058	141.432	712	34.067
2	3	0.156	126.809	120	5.742
3	1	0.401	90.208	127	6.077
4	2	0.568	65.188	114	5.455
5	11	0.927	11.512	1017	48.660
C-MP5					
1	12	0.047	143.123	1163	22.539
2	4	0.157	126.640	158	3.062
3	5	0.250	112.693	386	7.481
4	3	0.457	81.755	532	10.310
5	3	0.601	60.201	217	4.205
6	4	0.769	35.011	256	4.961
7	23	0.934	10.413	2448	47.442
C-MP10					
1	1	0.063	140.672	20	0.767
2	5	0.258	111.594	450	17.268
3	1	0.458	81.586	22	0.844
4	3	0.594	61.299	198	7.598
5	2	0.682	48.029	158	6.063
6	3	0.737	39.829	193	7.406
7	16	0.912	13.710	1565	60.054
MP					
1	14	0.085	141.432	1218	22.295
2	18	0.235	114.975	1509	27.622
3	15	0.281	108.128	1013	18.543
4	5	0.637	54.875	727	13.308
5	8	0.829	26.051	996	18.232

CON—control sample, C-MP2—sample with 2% mealworm powder addition, C-MP5—sample with 5% mealworm powder addition, C-MP10—sample with 10% mealworm powder addition, MP—mealworm powder.

**Table 3:** Nutritional value of crackers (%).

	Protein	Fat	Ash	Carbohydrates	Moisture	Energy kcal/100 g
CON	13.10 ± 0.01 ^d^	14.56 ± 0.95 ^c^	1.33 ± 0.13 ^b^	67.26 ± 1.12 ^a^	3.75 ± 0.75 ^b^	452 ± 4.07 ^c^
C-MP2	13.29 ± 0.12 ^c^	19.36 ± 0.20 ^b^	1.60 ± 0.06 ^a^	62.41 ± 1.50 ^b^	3.33 ± 0.10 ^b^	477 ± 4.86 ^b^
C-MP5	13.88 ± 0.07 ^b^	21.23 ± 0.85 ^b^	1.70 ± 0.09 ^a^	58.75 ± 1.74 ^b^	4.42 ± 0.23 ^a^	482 ± 4.07 ^ab^
C-MP10	15.36 ± 0.01 ^a^	23.42 ± 0.94 ^a^	1.73 ± 0.01 ^a^	54.62 ± 1.32 ^c^	4.87 ± 0.26 ^a^	491 ± 3.12 ^a^
MP	54.26 ± 0.12	31.74 ± 0.20	3.7 ± 0.05	2.345 ± 1.32	7.96 ± 0.96	512 ± 4.10

Values with different letters within the same column are significantly different (*p* ≤ 0.05). CON—control sample, C-MP2—sample with 2% mealworm powder addition, C-MP5—sample with 5% mealworm powder addition, C-MP10—sample with 10% mealworm powder addition.

**Table 4:** Mineral composition of crackers.

	Minerals (mg/100 g)						
	Ca	Mg	K	Na	Fe	Zn	Cu
CON	39.70 ± 6.27 ^a^	19.47 ± 0.69 ^c^	142.54 ± 0.00 ^c^	468.62 ± 5.52 ^a^	1.55 ± 0.06 ^c^	0.74 ± 0.13 ^b^	0.15 ± 0.04 ^b^
C-MP2	41.50 ± 1.94 ^a^	21.30 ± 0.44 ^bc^	150.34 ± 0.55 ^c^	467.85 ± 2.77 ^a^	1.62 ± 0.07 ^c^	0.75 ± 0.09 ^b^	0.15 ± 0.01 ^b^
C-MP5	42.87 ± 3.82 ^a^	25.03 ± 2.56 ^b^	166.97 ± 8.18 ^b^	465.20 ± 1.36 ^a^	1.85 ± 0.04 ^b^	1.07 ± 0.07 ^ab^	0.14 ± 0.06 ^b^
C-MP10	46.55 ± 6.20 ^a^	33.80 ± 2.11 ^a^	196.07 ± 3.57 ^a^	467.20 ± 0.00 ^a^	2.05 ± 0.03 ^a^	1.83 ± 0.67 ^a^	0.25 ± 0.00 ^a^
MP	75.50 ± 2.12	232.75 ± 6.01	865.00 ± 7.07	157.00 ± 1.41	3.74 ± 0.10	14.68 ± 0.04	1.43 ± 0.04

Values with different letters within the same column are significantly different (*p* ≤ 0.05). CON—control sample, C-MP2—sample with 2% mealworm powder addition, C-MP5—sample with 5% mealworm powder addition, C-MP10—sample with 10% mealworm powder addition.

**Table 5:** Antioxidant properties of crackers—antiradical activity and total phenolic content (TPC).

	ABTS+· (mM TEAC/g)	DPPH· (mM TEAC/g)	TPC (mg GAE/100 g)
CON	0.173 ± 0.03 ^c^	0.109 ± 0.01 ^c^	35.82 ± 0.69 ^d^
C-MP2	0.21 ± 0.02 ^c^	0.116 ± 0.05 ^c^	45.25 ± 0.68 ^c^
C-MP5	0.284 ± 0.03 ^b^	0.161 ± 0.07 ^b^	51.02 ± 0.92 ^b^
C-MP10	0.469 ± 0.07 ^a^	0.32 ± 0.09 ^a^	110.57 ± 1.41 ^a^
MP	1.31 ± 0.30	2.11 ± 0.20	418.7 ± 2.12

Values with different letters within the same column are significantly different (*p* ≤ 0.05). CON—control sample, C-MP2—sample with 2% mealworm powder addition, C-MP5—sample with 5% mealworm powder addition, C-MP10—sample with 10% mealworm powder addition, TEAC—Trolox Equivalent Antioxidant Activity, GAE—Gallic Acid Equivalents.

**Table 6:** Retention times (R_t_) and qualitative assessment of signal intensities of phenolic compounds identified in the control sample, *Tenebrio molitor* powder, and the product enriched with 10% *T. molitor* powder.

Substance	Rt (min)	Control Sample	*T. molitor* Powder	Product Enriched with 10% *T. molitor* Powder
Protocatechuic acid	11.051	±	+++	++
Caffeic acid	12.996	±	++	+
Chlorogenic acid	12.075	±	±	-
p-coumaric acid	14.229	±	±	±
Ferulic acid	14.693	++	+	+
Vanillic acid	13.143	+	±	±
Syringic acid	13.210	±	±	±
4-hydroxybenzoic acid	12.461	-	++	+
Protocatechuic acid aldehyde	12.461	+	+++	++
4-hydroxyacetophenone *	-	-	+++	++
Luteolin	18.802	-	++	-
Rutin	13.297	±	-	-
Apigenin-7-O-glucoside	14.386	+	±	-

(-) not detected; (±) trace level/very low signal intensity; (+) low signal intensity; (++) moderate signal intensity; (+++) high signal intensity, *—tentatively assigned based solely on characteristic MRM transitions (no authentic reference standard was available).

**Table 7:** Starch digestibility and predicted glycemic index.

	RDS (mg/g)	SDS (mg/g)	pGI
CON	139.48 ± 3.16 ^a^	101.28 ± 1.35 ^d^	56.32 ± 0.35 ^a^
C-MP2	116.36 ± 5.11 ^b^	109.47 ± 2.36 ^c^	55.84 ± 0.10 ^a^
C-MP5	110.32 ± 3.42 ^b^	125.25 ± 3.18 ^b^	54.94 ± 0.07 ^b^
C-MP10	93.44 ± 3.81 ^c^	132.31 ± 2.25 ^a^	54.82 ± 0.09 ^b^

Values with different letters within the same column are significantly different (*p* ≤ 0.05). CON—control sample, C-MP2—sample with 2% mealworm powder addition, C-MP5—sample with 5% mealworm powder addition, C-MP10—sample with 10% mealworm powder addition.

**Table 8:** Five-point hedonic scale consumer acceptance analysis and ranking method of consumer preferences.

		CON	C-MP2	C-MP5	C-MP10	χ^2^ Friedman	df	*p*	Kendall’s W
Color	mean ± SD	4.33 ± 1.13 ^a^	3.94 ± 0.90 ^b^	3.46 ± 0.95 ^c^	2.73 ± 1.55 ^d^	57.112	3	<0.001	0.307
	median (Q1–Q3)	5.0 (4.0–5.0)	4.0 (4.0–4.75)	3.5 (3.0–4.0)	3.0 (1.0–4.0)				
Crispness	mean ± SD	3.81 ± 1.49 ^a^	4.05 ± 0.94 ^a^	3.71 ± 0.98 ^a^	3.85 ± 1.10 ^a^	4.774	3	0.189	0.026
	median (Q1–Q3)	4.0 (3.0–5.0)	4.0 (3.0–5.0)	4.0 (3.0–4.75)	4.0 (3.0–5.0)				
Smell	mean ± SD	3.05 ± 0.97 ^a^	3.85 ± 0.97 ^b^	3.35 ± 1.18 ^a^	3.79 ± 1.06 ^b^	18.097	3	<0.001	0.097
	median (Q1–Q3)	3.0 (2.0–4.0)	4.0 (4.0–4.0)	3.0 (2.0–4.0)	4.0 (3.0–5.0)				
Taste	mean ± SD	3.72 ± 1.07 ^ab^	3.85 ± 0.99 ^a^	3.38 ± 1.21 ^a^	3.10 ± 1.06 ^b^	15.458	3	0.001	0.083
	median (Q1–Q3)	4.0 (3.0–5.0)	4.0 (3.0–4.75)	3.0 (2.0–4.0)	3.0 (2.0–4.0)				
Overall impression	mean ± SD	4.02 ± 0.97 ^a^	4.29 ± 0.74 ^a^	3.60 ± 0.89 ^b^	3.10 ± 1.10 ^b^	37.370	3	<0.001	0.201
	median (Q1–Q3)	4.0 (4.0–5.0)	4.0 (4.0–5.0)	4.0 (3.0–4.0)	3.0 (3.0–4.0)				
Ranking		110	132	91	62				

Values with different letters within the same row are significantly different. CON—control sample, C-MP2—sample with 2% mealworm powder addition, C-MP5—sample with 5% mealworm powder addition, C-MP10—sample with 10% mealworm powder addition.

**Table 9:** Formulation of control and mealworm powder-enriched crackers.

	CON	C-MP2	C-MP5	C-MP10
Wheat flour (g)	500	490	475	450
Egg (g)	65	65	65	65
Sugar (g)	15	15	15	15
Baking powder (g)	5	5	5	5
Salt (g)	4	4	4	4
Rapeseed oil (g)	85	85	85	85
Water (g)	85	85	85	85
Mealworm powder (g)	–	10	25	50

## Data Availability

The original contributions presented in the study are included in the article; further inquiries can be directed to the corresponding author.

## Supplementary Materials

The following supporting information can be downloaded at: https://www.mdpi.com/article/10.3390/ijms27209378/s1.

## Abbreviations

The following abbreviations are used in this manuscript:

ΔE	color difference
ABTS^+^·	2,20-azino-bis(3-ethylbenzothiazoline-6-sulfonic acid) radical
BI	browning index
CON	control cracker
DPPH·	2,2-diphenyl-1-picrylhydrazyl radical
FTIR	Fourier-transform infrared
GAE	gallic acid equivalents
GOPOD	glucose oxidase/peroxidase
MP	mealworm powder
pGI	predicted glycemic index
C-MP2	cracker with 2% mealworm powder
C-MP5	cracker with 5% mealworm powder
C-MP10	cracker with 10% mealworm powder
RDS	rapidly digestible starch
SDS	slowly digestible starch
TEAC	Trolox Equivalent Antioxidant Activity
