# Supplementary material for: Bioactive and Molecular Characterization of Mealworm (Tenebrio molitor) Powder: Nutritional and Functional Implications for Crackers
Source: Int J Mol Sci. 2026 Oct 21;27(20):9378. doi: 10.3390/ijms27209378 (PMC13705389; doi:10.3390/ijms27209378)
Supplement: Supplementary file 1 [file ijms-27-09378-s001.zip › ijms-4545674-supplementary.pdf]

International Journal of
Molecular Sciences

MDPI

Table S1. Multiple reaction monitoring (MRM) transitions.

Substance	Prec. Ion (m/z)	Prod. Ion (m/z)	Frag. (V)	CE (eV)
Gallic acid	169	125/79	130	15
Protocatechuic acid	153	109/91	130	15
Caffeic acid	179	134.9/107	130	20
Chlorogenic acid	353	191/179	130	18/20
p-coumaric acid	163	119/93	130	20
Ferulic acid	193	134/178	130	20
Vanillic acid	167	108/152	130	15
Syringic acid	197	182/123	130	15/25
Sinapic acid	223	208/164	130	15/25
4-hydroxybenzoic acid	137	93/65	130	15/25
Protocatechuic acid aldehyde	137	108/92	130	15/20
4-hydroxyacetophenone	135	92/65	130	15/25
Luteolin	285	133/151	130	25
Apigenin	269	117/151	130	25
Naringenin	271	151/119	130	25
Quercetin	301	151/179	130	30
Kaempferol	285	93/117	130	25
Catechin	289	245/205	130	25
Epicatechin	289	245/205	130	25
Rutin	609	301/463	130	30/20
Isoquercitrin	463	301/300	130	25/30
Hyperoside	463	301/300	130	25/30
Apigenin-7-O-glucoside	431	269/268	130	25
Naringenin-7-O-glucoside	433	271/151	130	25
Resveratrol	227	185/143	130	25
